# Antibiotic Overuse for COVID-19: Are We Adding Insult to Injury?

**DOI:** 10.4269/ajtmh.21-0603

**Published:** 2021-10-29

**Authors:** Seid Getahun Abdela, Laurens Liesenborghs, Fentaw Tadese, Seid Hassen Abegaz, Fentaw Bialfew Bayuh, Etsegent Arega Asmamaw, Tamiru Assefa Mebrate, Abreham Eshetu Mamo, Wendemaegn Embiale, Samuel Hunegnaw, Dereje Bedanie Hundie, Zewdu Hurissa, Gebi Agero, Abdene Weya Kaso, Maraki Assefa Mebrate, Saskia van Henten, Johan van Griensven

**Affiliations:** ^1^ College of Medicine and Health Sciences, Wollo University, Dessie, Ethiopia;; ^2^ Boru Meda General Hospital, Dessie, Ethiopia;; ^3^ Saint Peter Specialized Hospital, Addis Ababa, Ethiopia;; ^4^ College of Medicine and Health Sciences, Bahir Dar University, Bahir Dar, Ethiopia;; ^5^ Department of dermatology, Amsterdam Institute for Infection and Immunity (AI and II), Amsterdam UMC, University of Amsterdam, Amsterdam, The Netherlands;; ^6^ College of Health Sciences, Arsi University, Asella, Ethiopia;; ^7^ College of Health Sciences, Dilla University, Dilla, Ethiopia;; ^8^ Ethiopian Airlines Medical Unit Services;; ^9^ Department of Clinical Sciences, Institute of Tropical Medicine, Antwerp, Belgium

## Abstract

In this study, we described the proportion of COVID-19 patients started on antibiotics empirically and the work-ups performed to diagnose bacterial superinfection. We used a retrospective cohort study design involving medical records of symptomatic, hospitalized COVID-19 patients who were admitted to these centers. A total of 481 patients were included, with a median age of 41.0 years (interquartile range, 28-58.5 years). A total of 72.1% (*N* = 347) of COVID-19 patients received antibiotics, either before or during admission. This is troublesome because none of the patients’ bacterial culture or inflammatory markers, such as the erythrocyte sedimentation rate or C-reactive protein, were evaluated, and only 73 (15.2%) underwent radiological investigations. Therefore, national COVID-19 guidelines should emphasize the rational use of antibiotics for the treatment of COVID-19, a primarily viral disease. Integrating antimicrobial stewardship into the COVID-19 response and expanding microbiological capacities in low-income countries are indispensable. Otherwise, we risk one pandemic aggravating another.

Frequent and often inappropriate antibiotic use is common in low- and middle-income countries.^1^ Because of the worldwide increase in antimicrobial resistance, this is concerning. Moreover, we fear that the emergence of COVID-19 could further fuel the antimicrobial resistance pandemic. The role of antibiotics in the treatment of COVID-19 is not clearly defined. Many national and international guidelines, including Ethiopia’s, recommend the use of broad-spectrum antibiotics for patients with COVID-19 to treat any bacterial superinfection.^2^ Yet, the limited available evidence suggests that a bacterial superinfection is not a prominent feature of COVID-19.^3^ Nevertheless, antibiotic consumption is high among COVID-19 patients in most high-income settings^4^^,^^5^; however, it has not been assessed in African settings, where the diagnosis of bacterial coinfection is often difficult because of limited investigation modalities. We studied the antibiotic use by COVID-19 patients admitted to four Ethiopian treatment centers between May and November 2020, and assessed the work-up they underwent to diagnose bacterial superinfection.

The management of COVID-19 at the treatment centers is in line with the national treatment guidelines. Patients with an uncomplicated upper respiratory tract viral infection and nonspecific symptoms such as fever, fatigue, cough, or headache are considered to have a mild infection. Among adults, moderate illness is described as mild pneumonia according to the CURB-65 criteria (absence of confusion, urea, respiratory rate, and blood pressure, and age 65 years or older). Severe illness is described as severe pneumonia, acute respiratory distress syndrome, or sepsis responding to noninvasive management. Critical illness is considered when patients do not respond to noninvasive management, or when there is respiratory failure, septic shock, and/or multiple organ dysfunction or failure.^2^

A total of 481 patients with a median age of 41.0 years (interquartile range, 28–58.5) were included. The majority of patients were male (*N* = 342; 71.1%), and 190 (39.5%) had at least one comorbidity. More than half of them had mild cases (*N* = 242; 50.3%), 78 had moderate cases (16.2%), and 161 had severe or critical cases (33.5%) (Table 1). Comorbidities were documented among 190 patients (39.5%); hypertension (91; 47.9%) and diabetes mellitus (67; 35.3%) were the two most frequent comorbidities. Chronic respiratory conditions were reported among 23 patients (12.1%). The median hospital stay was 14 days (interquartile range, 10–17.5 days), and 58 patients (12%) died of COVID-19 complications.

**Table 1:** Antibiotics use before and after admission according to the severity status of COVID-19 patients treated across different treatment centers in Ethiopia in 2020

	Mild	Moderate	Severe and critical	Total
	*N* = 242	*N* = 78	*N* = 161	*N* = 481
Received antibiotics before admission	16 (2.5%)	25 (32.1%)	46 (28.6%)	87 (18.1%)
Oral route	6 (37.5%)	14 (56.0%)	11 (23.9%)	31 (35.6%)
Parenteral route	7 (43.8%)	6 (24.0%)	29 (63.0%)	42 (48.3%)
Both	3 (18.8%)	5 (20.0%)	6 (13.0%)	14 (16.1%)
Received antibiotics during admission	110 (45.5%)	73 (93.6%)	159 (98.8%)	342 (71.1%)
Oral route	81 (73.6%)	24 (32.9%)	3 (1.9%)	108 (31.6%)
Parenteral route	26 (23.6%)	23 (31.5%)	108 (67.9%)	157 (45.9%)
Both	3 (2.7%)	26 (35.6%)	48 (30.2%)	77 (22.5%)
Type of antibiotics				
Penicillin	42 (17.4%)	30 (38.5%)	19 (11.8%)	91 (18.9%)
Macrolides	77 (31.8%)	45 (57.7%)	51 (31.7%)	173 (36.0%)
Third-generation cephalosporin	33 (13.6%)	48 (61.5%)	135 (83.9%)	216 (44.9%)
Fourth-generation cephalosporin	0 (0%)	6 (7.7%)	26 (16.1%)	32 (6.7%)
Fluoroquinolones	3 (1.2%)	5 (6.4%)	8 (5.0%)	16 (3.3%)
Meropenem	0 (0%)	0 (0%)	20 (12.4%)	20 (4.2%)
Vancomycin	3 (1.2%)	22 (28.2%)	118 (73.3%)	143 (29.7%)
Investigations performed				
X-ray imaging	6 (2.5%)	13 (16.7%)	34 (21.1%)	53 (11.0%)
Chest computed tomography scan	0 (0.0%)	5 (6.4%)	15 (9.3%)	20 (4.2%)
Complete blood count	34 (14.0%)	39 (50.0%)	95 (59.0%)	168 (34.9%)

A total of 72.1% (*N* = 347) of COVID-19 patients received antibiotics, either before or during admission. Amoxicillin/clavulanate and azithromycin were the most used oral antibiotics, whereas ceftriaxone and vancomycin were the most frequently prescribed intravenous drugs. Nineteen patients (5.6%) received meropenem. This is troublesome because none of the patients’ bacterial culture or inflammatory markers, such as the erythrocyte sedimentation rate or C-reactive protein, were evaluated, and only 73 (15.2%) underwent radiological investigations.

National COVID-19 guidelines should emphasize the rational use of antibiotics for the treatment of COVID-19, which is a primarily viral disease. Antibiotics, especially broad-spectrum agents like meropenem, should be used only after careful consideration, and, ideally, they should be accompanied by bacterial culture and antimicrobial susceptibility tests. Integrating antimicrobial stewardship into the COVID-19 response and expanding microbiological capacities in low-income countries are indispensable. Otherwise, we risk one pandemic aggravating another.
